# Differential iridoid production as revealed by a diversity panel of 84 cultivated and wild blueberry species

**DOI:** 10.1371/journal.pone.0179417

**Published:** 2017-06-13

**Authors:** Courtney P. Leisner, Mohamed O. Kamileen, Megan E. Conway, Sarah E. O’Connor, C. Robin Buell

**Affiliations:** 1 Department of Plant Biology, Michigan State University, East Lansing, MI, United States of America; 2 Department of Biological Chemistry, The John Innes Centre, Norwich, United Kingdom; Wuhan Botanical Garden, CHINA

## Abstract

Cultivated blueberry (*Vaccinium corymbosum*, *Vaccinium angustifolium*, *Vaccinium darrowii*, and *Vaccinium virgatum*) is an economically important fruit crop native to North America and a member of the Ericaceae family. Several species in the Ericaceae family including cranberry, lignonberry, bilberry, and neotropical blueberry species have been shown to produce iridoids, a class of pharmacologically important compounds present in over 15 plant families demonstrated to have a wide range of biological activities in humans including anti-cancer, anti-bacterial, and anti-inflammatory. While the antioxidant capacity of cultivated blueberry has been well studied, surveys of iridoid production in blueberry have been restricted to fruit of a very limited number of accessions of *V*. *corymbosum*, *V*. *angustifolium* and *V*. *virgatum*; none of these analyses have detected iridoids. To provide a broader survey of iridoid biosynthesis in cultivated blueberry, we constructed a panel of 84 accessions representing a wide range of cultivated market classes, as well as wild blueberry species, and surveyed these for the presence of iridoids. We identified the iridoid glycoside monotropein in fruits and leaves of all 13 wild *Vaccinium* species, yet only five of the 71 cultivars. Monotropein positive cultivars all had recent introgressions from wild species, suggesting that iridoid production can be targeted through breeding efforts that incorporate wild germplasm. A series of diverse developmental tissues was also surveyed in the diversity panel, demonstrating a wide range in iridoid content across tissues. Taken together, this data provides the foundation to dissect the molecular and genetic basis of iridoid production in blueberry.

## Introduction

Iridoids are a large group of secondary metabolites found both in a variety of plant and selected animal species. They belong to the monoterpenes with a cyclopentan[c]pyran skeleton and occur in plant materials naturally as glucoside forms. Iridoids are abundant across several plant families including the Apocynaceae, Lamiaceae, Loganiaceae, Rubiaceae, Scrophulariaceae, and Verbenaceae [1]. The first steps in iridoid biosynthesis involve geraniol, which is hydroxylated by geraniol 8-hydroxylase/8-oxidase to form 8-hydroxygeraniol [2]. Subsequent oxidation, methylation and glycosylation steps form secologanin [2]. Genes for all steps in secologanin biosynthesis have been elucidated in the medicinal plant species, *Catharanthus roseus* [2].

In addition to their role in secondary plant metabolism, iridoids have known human health benefits including anti-inflammatory, anticancer, antimicrobial, antioxidant, antispasmodic, cardioprotective, choleretic, hepatoprotective, hypoglycemic, hypolipidemic, neuroprotective, and purgative activities [1,3,4]. For example, the iridoid compound acevaltrate isolated from the medicinal plant *Valeriana jatamansi* (syn. *V*. *wallichii*) was shown to have cytotoxic activity against several different cancer cell lines including adenocarcinoma, prostate cancer, colon cancer and hepatoma [5]. The iridoid compound phyloyoside I was isolated from rhizomes of the medicinal plant *Eremostachys laciniata* and demonstrated moderate antibacterial activity against five different bacterial strains [6]. Additionally, commercial production of supplements derived from Noni fruits (*Morinda citrifolia* Linn.), a tropical medicinal plant prized for its high levels of iridoid glycosides, began in the 1990s [7–8]. Most notably, the iridoid secologanin produced in *C*. *roseus* is used to synthesize vinblastine and vincristine, two potent anti-cancer alkaloids [2].

Iridoids also play a role in plant defense [9–10]. Iridoid glycosides are generally activated in the gut of insect herbivores by β-glucosidases that are co-ingested from the plant, or by endogenous insect β-glucosidases [9]. From this reaction, an unstable aglycone is released that can covalently cross-link with proteins, causing enzyme denaturation [9–12]. Therefore, iridoid glycosides act as a deterrent to non-adapted insects, as well as enforce specific adaptation in insect herbivores for host plants with these toxic specialized compounds [9].

Blueberries are within the Ericiaceae family and are represented by multiple species. The highbush (*V*. *corymbosum*) and lowbush (*V*. *angustifolium*) blueberry are the primary species of commercially grown blueberry. In regions with different horticultural requirements, stands of southern highbush (*V*. *corymbosum* with introgressions of *Vaccinium darrowii*) and rabbiteye (*V*. *virgatum*; syn. *V ashei*) are commercially grown. Blueberries can be grown for several different market classes including fresh market, processing and small-scale local production (farmers markets, pick-your-own). Fresh market and processing remain the largest market classes, with 8.1 million kg of organic fresh market blueberries and 2.5 million kg of organic processing blueberries purchased in the United States in 2014 [13]. In 2015, 36,349 hectares of blueberries were harvested in the United States, amounting to $5.84 billion in fresh market production and $2.27 billion in processing production [13]. Wild blueberries are also produced in the United States, with the largest harvested land found in Maine. A total of 9,065 hectares were harvested in 2015, producing an estimated 45.8 million kg of blueberries [13].

That majority of current blueberry breeding activity in the United States is focused on the northern and southern highbush and the rabbiteye ecotypes [14]. Current breeding efforts for southern highbush blueberry focus on early ripening, disease resistance, later flowering, higher yields and better flavor, while northern highbush blueberry breeding efforts are focused on flavor, longer fruit storage, expanded harvest dates, disease resistance and machine harvestability [14]. Rabbiteye blueberry breeders are focused on improving blueberry fruit quality and size, expanded harvest dates, longer storage life, and reducing susceptibility to rain cracking [14]. Introgression of wild germplasm has been used historically in blueberry breeding, especially in the development of the southern highbush blueberry to introduce traits such as disease resistance, low chilling, tolerance to drought, heat and mineral soils, and improved fruit color and flavor [15].

Blueberries are well known for containing health-promoting dietary bioactive compounds including folate, vitamin C, flavonoids and phenolic compounds [16]. Blueberries are also prized for their potent antioxidant capacity [17–19], attributable primarily to anthocyanins, procyanidins, chlorgenic acid and flavonoid compounds present in plant tissues [20]. The bioactive compounds in blueberry have also been shown to have many human health benefits including lowering blood pressure [21], inhibiting the growth of tumor cells [22–24], and potential prevention of neurodegenerative disease [25].

Iridoid compounds have been identified in a small number of species within the Ericaceae family (Table 1). Previous work reported the iridoid compound gardenoside in fruit tissue of neotropical blueberries [26], the iridoid glycoside monotropein in fruit juice of cranberry (American and European), lignonberry, and bilberry [27], and a compound similar to monotropein in green tissues, stems, and/or fruit of European cranberry, lingonberry, and bilberry [28] (Table 1). A recent survey of iridoid glycosides in four *Vaccinium* species found 14 different iridoid glycoside compounds in fruits and/or fruit juice of *V*. *uliginosum* (bog bilberry) and 11 different iridoid glycoside compounds in *V*. *myrtillus* (bilberry) [29] (Table 1). Surprisingly, although iridoids have been found in close relatives of cultivated blueberries relevant to North American blueberry production, no iridoid glycosides have been detected to date in *V*. *corymbosum*, *V*. *angustifolium*, or *V*. *virgatum* (Table 1). Previous work by Ma et al., (2013) [26] did not detect gardenoside in the fruit of any North American blueberry species (*V*. *corymbosum*, *V*. *angustifolium*, *V*. *virgatum*). Nor did Heffels et al., (2016) [29] detect any iridoid glycosides in the fruit of *V*. *corymbosum* (highbush blueberry), or *V*. *angustifolium* (lowbush blueberry). One limitation of all of these studies is that only a single cultivar of highbush blueberry was surveyed [26, 29], limiting the diversity of cultivated blueberry germplasm represented in previous work.

**Table 1:** Previously published research on iridoids in *Vaccinium* species and neotropical blueberries.

Species	Common name/Ecotype	Tissue	Presence of Iridoid compounds	Reference
*V*. *myrtillus*	Bilberry	Stem and fruit	Yes	28
		Fruit juice	Yes	27
		Fruit and fruit juice	Yes	29
*V*. *macrocarpon*	American Cranberry	Fruit juice	Yes	27
*V*. *oxycoccos*	European Cranberry	Green tissues	Yes	28
		Fruit juice	Yes	27
*V*. *vitis-idaea*	Lingonberry	Fruit juice	Yes	27
		Leaves and stems	Yes	28
*Cavendishia isernii*	Neotropical blueberry	Fruit	Yes	26
*Sphyrospermum ellipticum*	Neotropical blueberry	Fruit	Yes	26
*Macleania coccoloboides*	Neotropical blueberry	Fruit	Yes	26
*Macleania cordifolia*	Neotropical blueberry	Fruit	Yes	26
*Satyria boliviana*	Neotropical blueberry	Fruit	Yes	26
*V*. *uliginosum*	Bog bilberry	Fruit	Yes	29
*V*. *corymbosum* cv. Briggita	Northern highbush blueberry	Fruit	No	26
*V*. *corymbosum*	Northern highbush blueberry	Fruit	No	29
*V*. *angustifolium*	Lowbush blueberry	Fruit	No	26 29
*V*. *virgatum*	Rabbiteye blueberry	Fruit	No	26

To address this knowledge gap, we constructed a diversity panel composed of 13 wild blueberry species and 71 cultivated blueberry accessions (n = 84 total accessions) to provide a broad survey of the iridoid glycoside monotropein in cultivated blueberry fruits and leaves. Additionally, iridoid content was measured in a developmental tissue series from a subset of the diversity panel in the following year to determine the range of monotropein content across diverse developmental stages. Our discovery of monotropein in several cultivars and multiple tissues can facilitate our understanding of the biochemistry and synthesis of these potent natural products and more importantly, how their diversity arose in cultivated blueberry.

## Materials and methods

### Tissue sampling

In total, ripe fruit and young leaf tissue was collected for 71 cultivated blueberry varieties and 13 wild *Vaccinium* spp (listed in S1 Table). Commercial blueberries were collected from the Michigan Blueberry Growers Association (MBGA) in Grand Junction, MI (42°24'09.4"N 86°04'20.9"W). Permission for sampling was obtained prior to sample collection from Ed Wheeler, blueberry breeder at MBGA. Tissue samples for ripe fruit and young leaves were collected at the time of fruit ripening. Cultivars ripened at different rates, therefore sampling occurred over several weeks in July, 2015. Samples were harvested on site, placed on ice and transported to East Lansing, MI where they were flash frozen with liquid nitrogen and stored at -80°C. The following year (2016), a tissue series for a subset of monotropein-positive and–negative cultivars was collected from the same plants grown at MBGA. The cultivars included were Bluecrop, Cara’s Choice, Concord, Ornablue, Ozarkblue and Summit. These tissues included floral buds, young leaves, mature leaves, unripe fruit, ripe fruit and stems. Samples were harvested on site, placed on ice and transported to East Lansing, MI where they were flash frozen with liquid nitrogen and stored at -80°C.

Wild species were obtained from the USDA Germplasm Resources Information Network (GRIN), Corvallis OR. The wild species collected from USDA GRIN represent *Vaccinium* spp. from several different countries of origin. Accession identification information for all wild samples is located in S1 Table. Samples were shipped overnight on ice, and then flash frozen and stored at -80°C upon receipt. A single biological replicate was obtained for 13 wild *Vaccinium* spp. from USDA GRIN.

### Iridoid metabolite analysis and quantification

**Fig 1:**
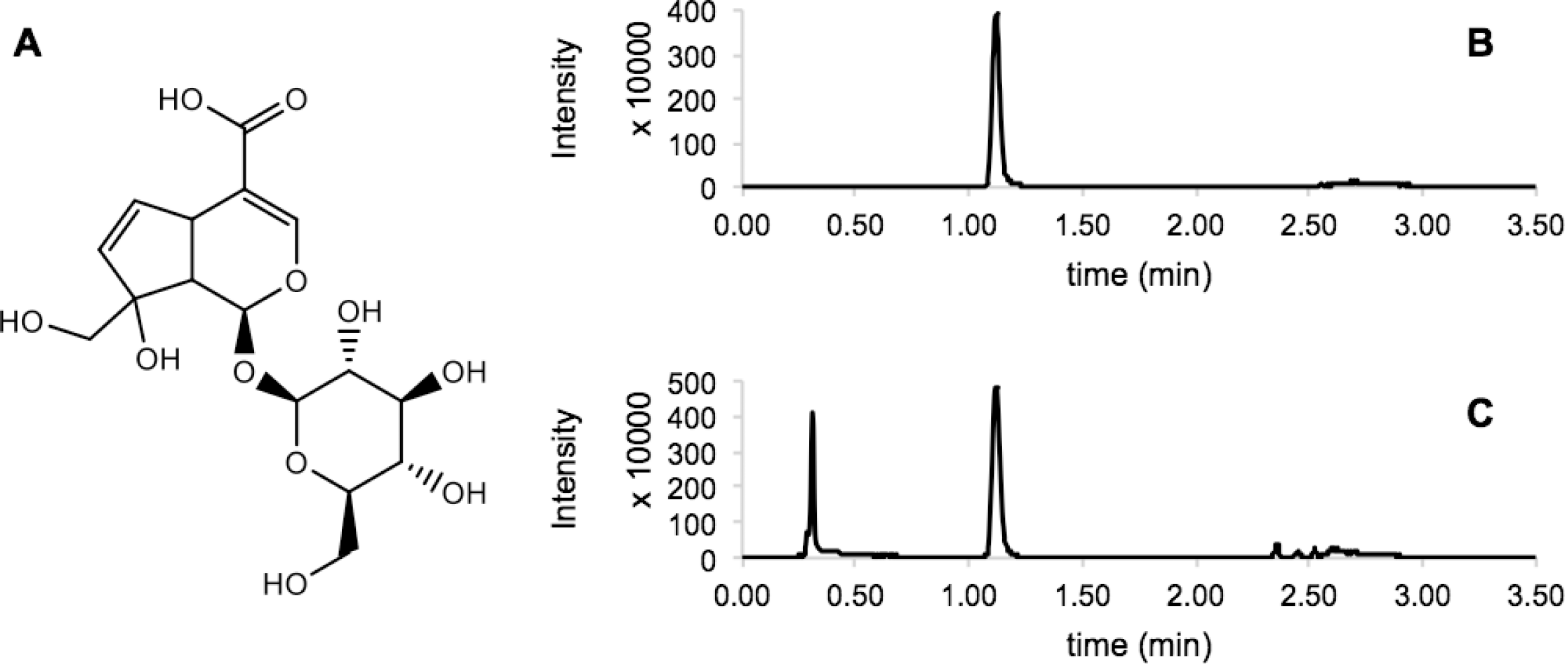
Example chromatogram demonstrating the presence of monotropein in blueberry. Chemical structure of iridoid glycoside monotropein (A). Multiple reaction monitoring (MRM) chromatograms (413.013 > 233.094) of analytical standard monotropein (B), alongside representative blueberry cultivar Summit (C). Monotropein was detected as a sodium adduct (*m/z* 413.013; [M+Na]^+^) in positive mode.

The iridoid glycoside monotropein (Fig 1) has been previously identified in other *Vaccinium* species within the Ericaceae [26–29] and was therefore used as a diagnostic marker to determine the presence/absence of iridoids in an 84-member diversity panel that represents wild and cultivated blueberries. Monotropein was analyzed and quantified by liquid chromatography mass spectrometry (LC-MS) using ground, lyophilized tissues. An authentic standard of monotropein was used for iridoid identification and quantification. An initial screen for monotropein was performed in berries from all 84 members of the diversity panel using a single biological replicate from the 2015 field season. For the five monotropein positive cultivars (Bluehaven, Blueridge, Ornablue, Ozarkblue, Summitt), quantification was then completed for two or three biological replicates of both young leaves and ripe fruit from the 2015 field season, with the exception of Summit, which had a single biological replicate. Floral buds, ripe fruit, unripe fruit, stems, immature leaves, and mature leaves were sampled from one or two biological replicates in the 2016 field season from three monotropein positive cultivars (Ornablue, Ozarkblue, Summit) along with three monotropein negative cultivars (Bluecrop, Cara’s Choice, and Concord).

#### Chemicals and reagents

Methanol (Analytical grade), formic acid (LC-MS grade), and acetonitrile (LC-MS grade) were purchased from Sigma Aldrich. Ultrapure water was produced by Milli-Q-System (Bedford, MA, USA). Iridoid glycoside standard monotropein (≥98% HPLC grade) was purchased from Sigma Aldrich (SMB00471). The standard was dissolved in distilled H_2_O (dH_2_O) to obtain a stock concentration of 100 μg/mL and was stored at -20°C.

#### Sample preparation

Lyophilized blueberry tissue samples (20 mg) were weighed and extracted with 400 μL of methanol. The extract was vortexed at 800 rpm for 10 min and placed at 60°C for 1 hour. After incubation, the extract was centrifuged and the supernatant was diluted 1:20 with distilled water (dH_2_O). The diluted extract was filtered through a 0.2 μm PTFE filter (Sigma Aldrich) before UPLC-QqQ-MS/MS analysis.

To initially test that no decomposition of the iridoid glycoside was occurring at higher temperatures, a known concentration (0.47 μM) of the monotropein standard (Sigma Aldrich) was incubated at 60°C for 1 hour in methanol and the chromatographic traces were compared to an identical sample that had been incubated at room temperature (S1 Fig). There was no difference in retention time, peak intensity, or formation of new peaks, indicating the stability of the glycosidic bond under these conditions.

To ensure that monotropein was stable in the presence of blueberry fruit extracts at 60°C, a blueberry cultivar (Summit) (which contains small amounts of monotropein) was spiked with a known concentration of monotropein standard (Sigma Aldrich). The sample was then extracted under the conditions described above and amount of monotropein that was recovered was quantified (S2 Table). An independent t-test showed there was no significant difference between the spiked and recovered concentration of monotropein in blueberry tissue (*p*-value, 0.189257; d.f., 4; *p* < 0.05), thus highlighting that the matrix has no substantial effect in hydrolyzing the glycosidic bond and confirmed the stability of iridoid glycosides under the extraction protocol described above.

#### UPLC-QqQ-MS/MS analysis

Sample analysis was carried out with an Acquity ultra-performance liquid chromatography (UPLC) system (Waters Corp., Milford, MA, USA) coupled to a Xevo TQ-S triple quadrupole tandem mass spectrometer equipped with an electrospray ionization interface (ESI) (Waters Corp., Milford, MA, USA). Chromatographic separation was performed using an Acquity UPLC BEH C18 column (2.1 mm x 50 mm; 1.7 μm particle size) (Waters Corporation, Milford, MA, USA). The column was kept at 40°C whilst the auto sampler was set at 10°C. The injection volume for both the solution of standards and analytes was 1 μL. The flow rate of the mobile phase was 0.5 mL/min. The mobile phase consisted of water containing 0.1% formic acid (eluent A) and acetonitrile (eluent B). A gradient elution was performed as follows; the proportion of eluent B was linearly increased from 0% to 5% in 1 min, then increased to 90% in 0.5 min and kept constant for 1 min. The column was re-equilibrated with 100% elutent A for 1 min before the next injection took place. The duration of each UPLC run was 4.6 min. Each wash cycle consisted of 200 μL strong solvent (acetonitrile + 0.01% formic acid) and 600 μL of weak solvent (10% acetonitrile + 0.01% formic acid). Mass spectra were acquired in positive electrospray ionization (ESI) mode. Capillary voltage was 2.5 kV; the source was kept at 150°C; desolvation temperature was 600°C; cone gas flow at 50 Lh^-1^; and desolvation gas flow at 900 Lh^-1^. Unit resolution was applied to each quadrupole. Targeted method for identification of monotropein was developed using a commercial standard (Sigma-Aldrich). Flow injection of monotropein was used to optimize the multiple reaction monitoring (MRM) conditions using Waters Intellistart software. Monotropein was detected as a sodium adduct (*m/z* 413.013; [M+Na]^+^) in ESI+ mode. Four MRM transitions were used to monitor the elution of monotropein. MRMs used for the detection of monotropein (ES+) were: *m/z* 413.013 > 185.77 (cone 32, collision 22), *m/z* 413.013 > 203.094 (cone 32, collision 22), *m/z* 413.013 > 233.074 (cone 32, collision 24), and *m/z* 413.013 > 251.089 (cone 32, collision 22). Transition *m/z* 413.013 > 233.074 (cone 32, collision 24) was used for quantification of monotropein.

Data acquisition was performed with TargetLynx 4.1 Waters Xevo TQ-S quantitative analysis software and data processing was executed using MassLynx 4.1 mass spectrometry software.

#### Limit of detection, limit of quantitation, and linearity

The limit of detection (LOD) and limit of quantitation (LOQ) for the presented MRM method was set at signal-to-noise ratios of (S/N) >3 and >10, respectively. For quantification of monotropein, a calibration curve was prepared from a stock solution of 5 mg/mL monotropein and analysed using ten calibrators diluted in dH_2_O in a range of 2.4 mg/mL to 4.7 ng/mL. The linearity acceptance criterion for the correlation coefficient (R^2^) was 0.99 or better.

## Results and discussion

### Survey of the iridoid glycoside monotropein in the blueberry diversity panel

**Fig 2:**
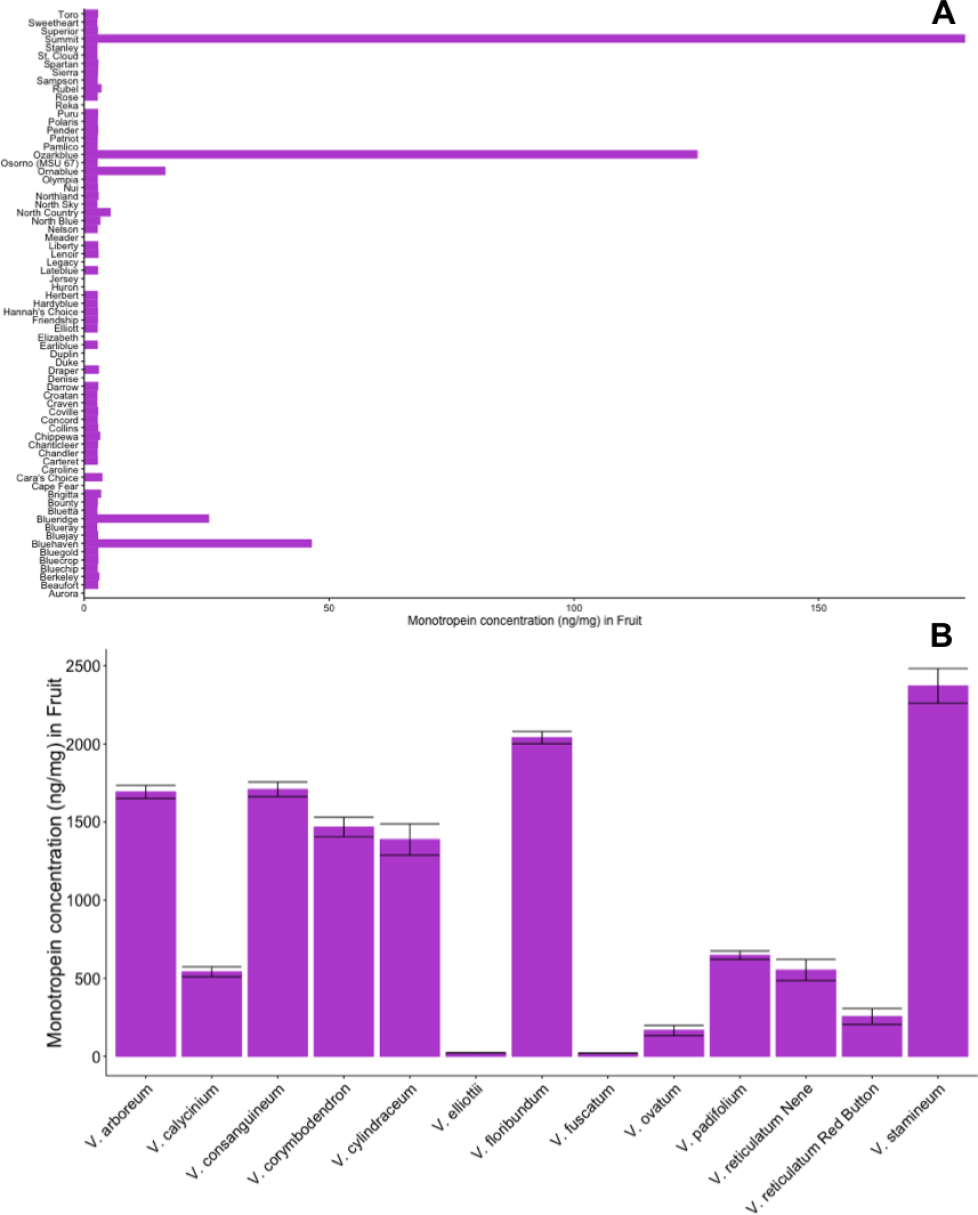
Quantification of the glycoside iridoid monotropein in fruit tissue for the 84-member blueberry diversity panel. A) Quantification of monotropein for all 71 cultivated blueberry varieties sampled in 2015. Values of zero represent cultivars where monotropein could not be detected under current conditions, i.e. below the limit of detection. B) Quantification of monotoropein for all 13 wild *Vaccinium* species. Error bars represent mean ± standard error (n = 1, samples analyzed in triplicate).

A survey of iridoid content in the 84-member diversity panel (71 cultivars and 13 wild species) revealed monotropein present in concentrations greater than 10 ng/mg dry weight in ripe fruit of only five of the 71 cultivars in the panel (S1 Table; Fig 2A). The cultivars that contained monotropein in ripe fruit were Bluehaven, Blueridge, Ornablue, Ozarkblue and Summit. Values of monotropein content in ripe fruit ranged from 2.5–179.9 ng/mg across all 71 cultivars where monotropein was above the level of detection. All of the 13 wild *Vaccinium* spp. in the diversity panel contained monotropein in ripe fruit (Fig 2B). Values of monotropein in wild species ranged from 20.3–2371.1 ng/mg. Overall, levels of monotropein in ripe fruits of wild *Vaccinium* species was ~ 11 times higher than in ripe fruit of monotropein-positive cultivar cultivars.

**Fig 3:**
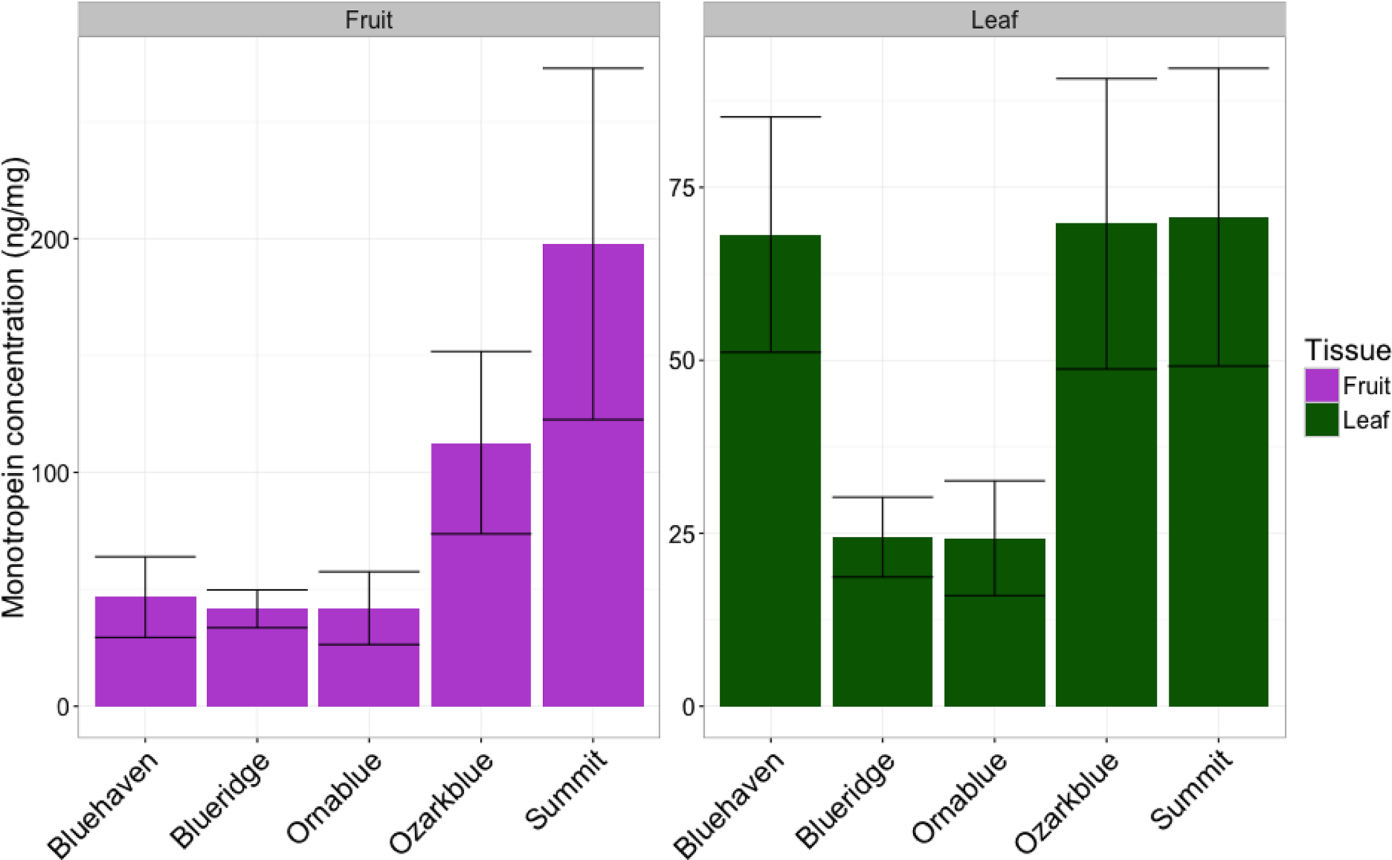
Quantification of the iridoid glycoside monotropein in ripe fruit and young leave tissue of five blueberry cultivars. Error bars represent the mean ± standard error (n = 1–3).

Further analysis of monotropein content in ripe fruit and young leaves was completed for the five monotropein-positive cultivars (Fig 3); all five cultivars which showed presence of monotropein in ripe fruit tissues also showed presence in young leaf tissue. Monotropein content of ripe fruit tissues ranged from 41–197 ng/mg, and 24–70 ng/mg in young leaf tissue (Fig 3; S1 Table). On average, there was approximately 1.7 times as much monotropein present in ripe fruit compared to young leaves in the monotropein-positive cultivars (Fig 3).

**Fig 4:**
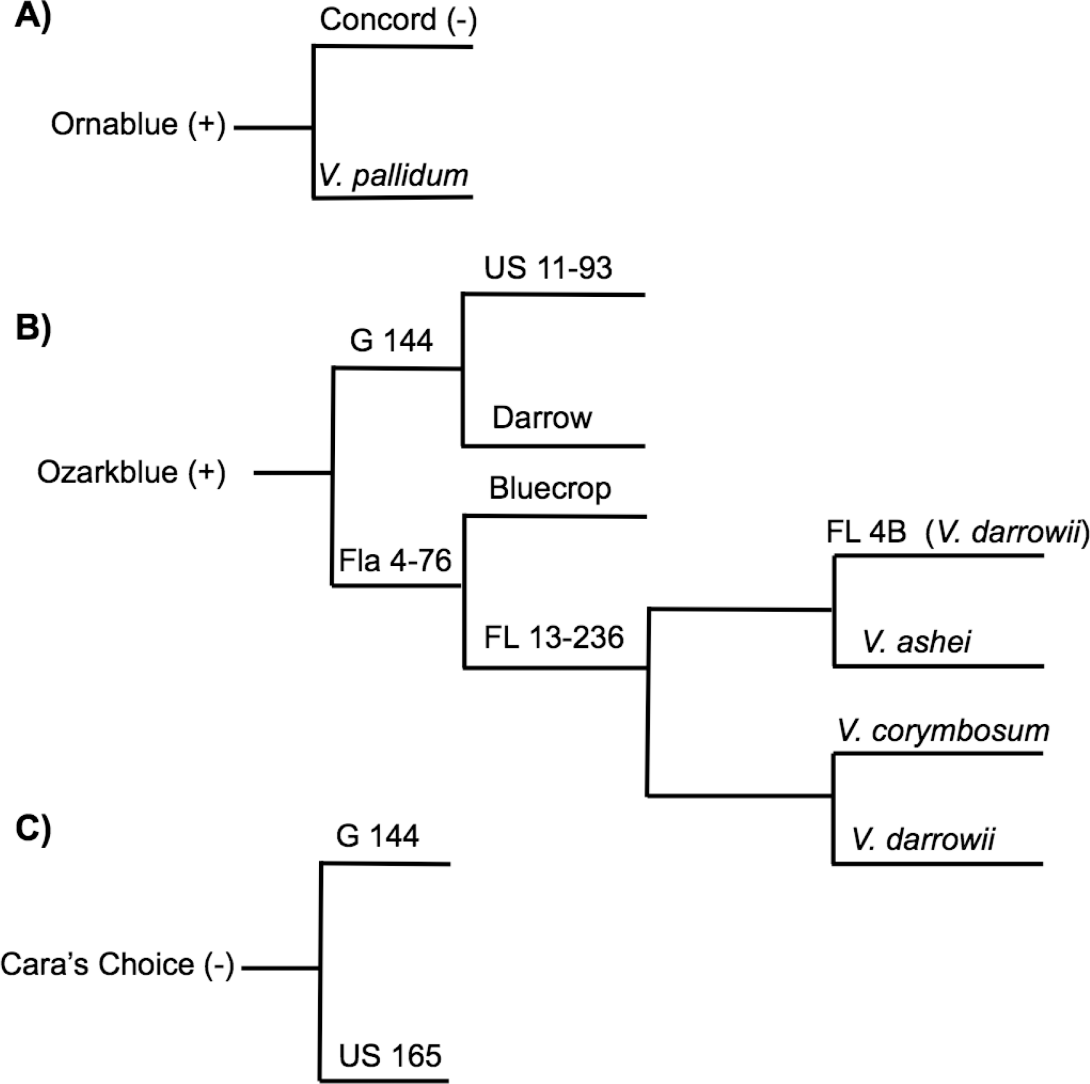
Pedigree diagram of select monotropein-positive and negative cultivars in blueberry diversity panel. A) Ornablue, B) Ozarkblue (Summit is a full-sibling and thus has the same pedigree), and C) Cara’s Choice. Ozarkblue pedigree information adapted from [30].

Ecotype and pedigree analysis of monotropein-positive and–negative cultivars for the subset of the panel was also completed. With the exception of Bluehaven, all monotropein-positive cultivars were either the southern highbush or half highbush ecotype (hybrids of highbush x lowbush) (Table 2; Fig 4). The majority of cultivated blueberries grown for fresh market in Michigan is the northern highbush ecotype, and thus was dominant in our diversity panel possibly explaining why monotropein was not identified in the majority of our diversity panel. Pedigree analysis of monotropein-positive and -negative cultivars from a subset of the panel revealed a prevalence of wild *Vaccinium* parentage in monotropein-positive cultivars (Table 2). The parents of Ornablue, a half-highbush blueberry cultivar, are the monotropein-negative cultivar Concord and *V*. *pallidum* (Table 2; Fig 4A). Ozarkblue and Summit, both monotropein-positive, are full siblings sharing G 144 and Fl 4–76 as parents [15] (Table 2; Fig 4B). Interestingly, the monotropein-negative cultivar Cara’s Choice is a half-sibling with Ozarkblue and Summit sharing G 144 as a parent [30–31] (Fig 4C). G 144 is a USDA northern highbush selection, while Fl 4–76 is an interspecific hybrid of *V*. *corymbosum*, *V*. *darrowi* and *V*. *virgatum* (syn. *V*. *ashei*) [31]. Due to the presence of monotropein in all wild *Vaccinium* species in this diversity panel, and prevalence of wild *Vaccinium* parentage in monotropein-positive cultivars, we hypothesize the presence of monotropein in these cultivars is due to introgressions from wild species into cultivated blueberry.

**Table 2:** Cultivar, ecotype and pedigree information for five monotropein-positive cultivars from the blueberry diversity panel.

Cultivar	Parent 1	Parent 2	Ecotype
**Bluehaven^*a*^**	Berkeley	19-H	NH
**Blue Ridge^*a*^**	Patriot	US 74	SH
**Ornablue^*a*^**	Concord	*V*. *pallidum*	HH
**Ozarkblue^*a*^**	G 144	Fla 4–76	SH
**Summit^*a*^**	G 144	Fla 4–76	SH

^*a*^Represents cultivars with monotropein present in ripe fruit and young leaves. NH = northern highbush; SH = southern highbush; HH = half highbush.

### Quantification of the iridoid glycoside monotropein in the blueberry tissue panel

**Fig 5:**
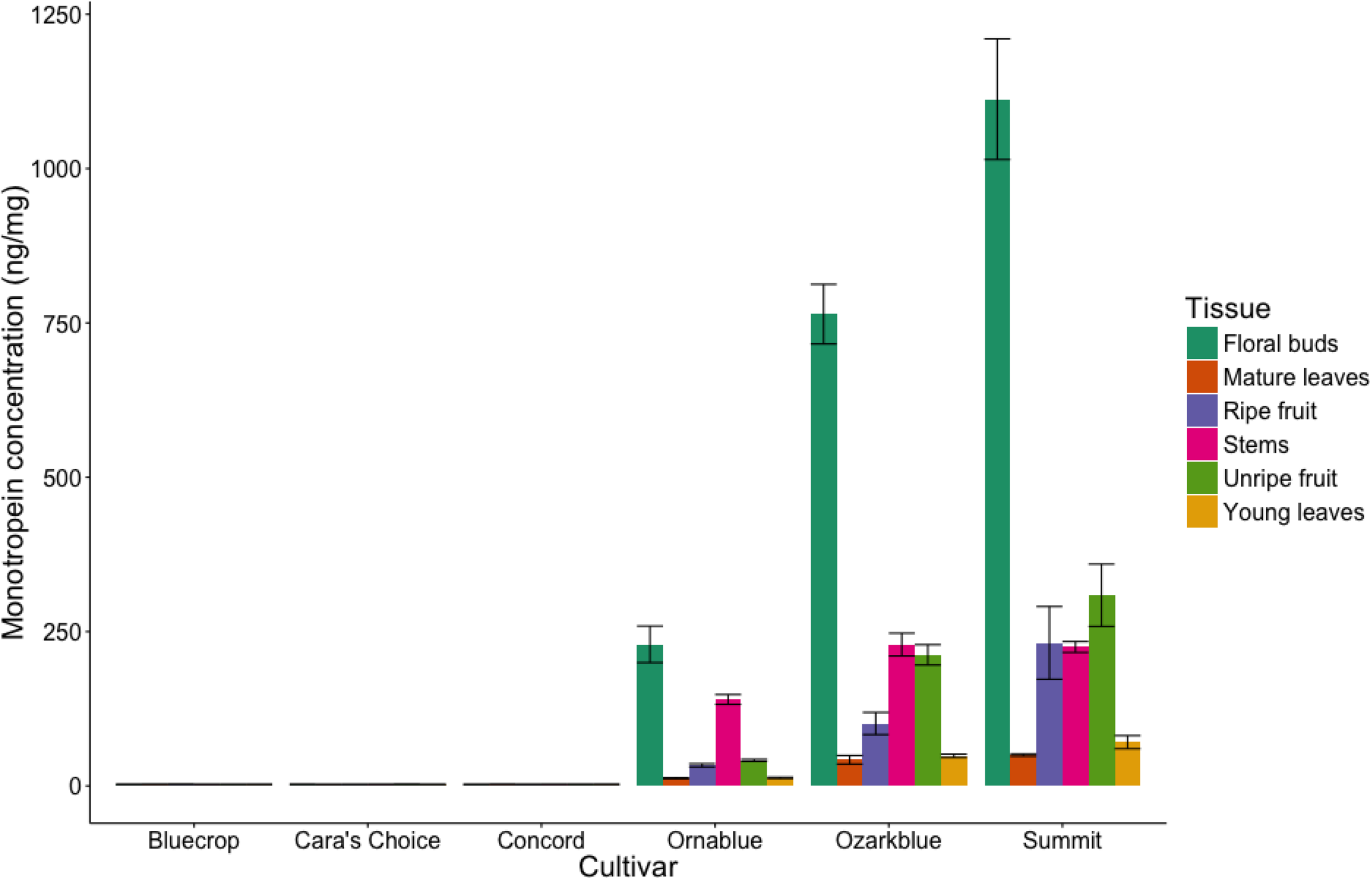
Quantification of the iridoid glycoside monotropein in a tissue series for six cultivars from the blueberry diversity panel. Error bars represent mean ± standard error (n = 1 or 2).

In order to quantify the full range of iridoid content in blueberry, a tissue series for six monotropein-positive and–negative cultivars was sampled the following year (2016). Six tissue types (floral buds, young leaves, mature leaves, stems, unripe fruit and ripe fruit) from three monotropein-negative cultivars (Bluecrop, Cara’s Choice, Concord) and three monotropein-positive cultivars (Ornablue, Ozarkblue, Summit) were collected and targeted LC-MS analysis was performed to detect and quantify monotropein (Fig 5). Cultivars identified as monotropein-negative in 2015 were also monotropein-negative in 2016 in all tissues sampled. Conversely, all cultivars identified as monotropein-positive in 2015 were monotropein-positive for all tissues samples in 2106.

In monotropein-positive cultivars, monotropein content ranged from 12.4–1112.5 ng/mg among all tissues (S3 Table). Additionally, across all three monotropein-positive cultivars the highest monotropein content was found in floral buds, with Summit showing the highest measured value (Fig 5; S3 Table). On average, there was approximately 5.9 times as much monotropein in floral buds versus the average monotropein content across all other tissues in monotropein-positive cultivars (S3 Table). Due to the role iridoid glucosides play in secondary defense [9–10], we hypothesize that the high monotropein content in floral tissue could be involved in plant insect defense.

## Conclusion

This study evaluated the presence of iridoid glycosides, potent natural products with potential human health benefits, in fruits of a diversity panel of 84 blueberry cultivars and wild species, the largest panel of cultivated blueberries surveyed to date. Data generated from this study revealed the presence of the iridoid glycoside monotropein in ripe fruit and young leaves of a limited number of cultivars of southern highbush and half highbush ecotypes, but only one cultivar of the common commercial northern highbush ecotype. Based on available pedigree data, we hypothesize that the presence of iridoid glycosides in cultivated blueberry is due to introduction of genes for iridoid biosynthesis, or regulation of biosynthetic pathway genes from wild species, thereby providing a path for targeted breeding of iridoid producing blueberry cultivars. Finally, data from this study identified floral bud tissues as containing the highest levels of monotropein, potentially due to their role in plant insect defense. Overall, findings from this study can be used to further our understanding of human health benefits of blueberry and how to exploit natural plant products in this cultivated fruit crop.

## Supporting information

S1 Fig Effect of monotropein under thermal treatment. Total ion chromatograms (TICs) of iridoid glycoside, monotropein after one hour incubation in methanol at room temperature (A) and 60°C (B).
(TIFF)

S1 Table Complete member list of blueberry diversity panel (sampled in 2015). (XLSX)

S2 Table The recovery of monotropein standard from a blueberry sample. (DOCX)

S3 Table Monotropein concentration for tissue panel from subset of blueberry cultivars (sampled in 2016). (XLSX)

## References

[1] Viljoen A, Mncwangi N, Vermaak I. Anti-inflammatory iridoids of botanical origin. Curr Med Chem. 2012; 19: 2104–2127. doi: 10.2174/092986712800229005 22414102 10.2174/092986712800229005 PMC3873812

[2] Miettinen K, Dong L, Navrot N, Schneider T, Burlat V, Pollier J, et al The seco-iridoid pathway from *Catharanthus roseus*. Nat Commun. 2014; 5: 3606–3616. doi: 10.1038/ncomms4606 24710322 10.1038/ncomms4606 PMC3992524

[3] Tundis R, Loizzo MR, Menichini F, Statti GA, Menichini F. Biological and pharmacological activities or iridoids: Recent developments. Mini Rev Med Chem. 2008; 8: 399–420. 18473930 10.2174/138955708783955926

[4] Dinda B, Debnath S, Banik R. Naturally occurring iridoids and secoiridoids. An updated review, part 4. Chem Pharm Bull. (Tokyo). 2011; 59: 803–833. 21720031 10.1248/cpb.59.803

[5] Lin S, Shen Y-H, Li H-L, Yang X-W, Chen T, Lu L-H, et al Acylated iridoids with cytotoxicity from *Valeriana jatamansi*. J Nat Prod. 2009; 72: 650–655. doi: 10.1021/np800716f 19245261 10.1021/np800716f

[6] Modaressi M, Delazar A, Nazemiyeh H, Fathi-Azad F, Smith E, Rahman MM, et al Antibacterial iridoid glucosides from *Eremostachys laciniata*. Phyther Res. 2009; 23: 99–103. 10.1002/ptr.2568 18693303

[7] Potterat O, Hamburger M. *Morinda citrifolia* (Noni) fruit–phytochemistry, pharmacology, safety. Planta Med 2007; 73: 191–199. doi: 10.1055/s-2007-967115 17286240 10.1055/s-2007-967115

[8] Deng S, West BJ, Palu AK, Jensen CJ. Determination and comparative analysis of major iridoids in different parts and cultivation sources of *Morinda citrifolia*. Phytochem Anal. 2011; 22: 26–30. doi: 10.1002/pca.1246 20799271 10.1002/pca.1246

[9] Dobler S, Petschenka G, Pankoke H. Coping with toxic plant compounds- The insect’s perspective on iridoid glycosides and cardenolides. Phytochemistry. 2011; 72: 1593–1604. doi: 10.1016/j.phytochem.2011.04.015 21620425 10.1016/j.phytochem.2011.04.015

[10] Pentzold S, Zagrobelny M, Rook F, Bak S. How insects overcome two-component plant chemical defense: plant β-glucosidases as the main target for herbivore adaptation. Biol Rev. 2014; 89: 531–551. 25165798 10.1111/brv.12066

[11] Kim D-H, Kim B-R, Kim J-Y, Jeong YC. Mechanism of covalent adduct formation of acubin to proteins. Toxicol Lett. 2000; 114: 181–188. 10713483 10.1016/s0378-4274(99)00295-7

[12] Konno K, Hirayama C, Yasui H, Nakamura M. Enzymatic activation of oleuropein: A protein crosslinker used as a chemical defense in the privet tree. Proc Natl Acad Sci USA. 1999; 96: 9159–9164. 10430912 10.1073/pnas.96.16.9159 PMC17749

[13] USDA National Agricultural Statistics Service. (2016), https://quickstats.nass.usda.gov, Accessed August 29, 2016.

[14] Hancock JF, Lyrene P, Finn CE, Vorsa N, Lobos GA. Blueberres and Cranberries In: Temperate Fruit Crop Breeding, Hancock J.F., Eds., Springer: Netherlands; 2008 pp. 115–149.

[15] Brevis PA, Bassil NV, Ballington JR, Hancock JF. Impact of wide hybridization on highbush blueberry breeding. J Amer Soc Hort Sci. 2008; 133: 427–437.

[16] Wang H, Guo X, Hu X, Li T, Fu X, Liu RH. Comparison of phytochemical profiles, antioxidant and cellular antioxidant activities of different varieties of blueberry (*Vaccinium* spp.). Food Chemistry. 2017; 217: 773–781. doi: 10.1016/j.foodchem.2016.09.002 27664697 10.1016/j.foodchem.2016.09.002

[17] Bunea A, Rugina D, Sconta Z, Pop RM, Pintea A, Socaciu C, et al Anthocyanin determination in blueberry extracts from various cultivars and their antiproliferative and apoptotic properties in B16-F10 metastatic murine melanoma cells. Phytochemistry. 2013; 95: 436–444. doi: 10.1016/j.phytochem.2013.06.018 23890760 10.1016/j.phytochem.2013.06.018

[18] Guerra MC, Galvano F, Bonsi L, Speroni E, Costa S, Renzulli C, et al Cyanidin-3-*O*-β-glucopyranoside, a natural free-radical scavenger against aflatoxin B1- and ochratoxin A-induced cell damage in a human hepatoma cell line (Hep G2) and a human colonic adenocarcinoma cell line (CaCo-2). Br J Nutr. 2005; 94: 211–220. 16115355 10.1079/bjn20051425

[19] Manganaris GA, Goulas V, Vicente AR, Terry LA. Berry antioxidants: small fruits providing large benefits. J Sci Food Agric. 2013; 94: 825–833. doi: 10.1002/jsfa.6432 24122646 10.1002/jsfa.6432

[20] Moyer RA, Hummer KE, Finn CE, Frei B, Wrolstad RE. Anthocyanins, phenolics, and antioxidant capacity in diverse small fruits: *Vaccinium*, *Rubus*, and *Ribes*. J Agric Food Chem. 2002; 50: 519–525. 11804523 10.1021/jf011062r

[21] Shaughnessy KS, Boswall IA, Scanlan AP, Gottschall-Pass KT, Sweeney MI. Diets containing blueberry extract lower blood pressure in spontaneously hypertensive stroke-prone rats. Nutr Res. 2009; 29: 130–138. doi: 10.1016/j.nutres.2009.01.001 19285604 10.1016/j.nutres.2009.01.001

[22] Liu J, Zhang W, Jing H, Popovich DG. Bob bilberry (*Vaccinium uliginosum* L.) extract reduces cultured Hep-G2, Caco-2, and 3T3-L1 cell viability, affects cell cycle progression, and has variable effects on membrane permeability. J Food Sci. 2010; 75: 103–107. 10.1111/j.1750-3841.2010.01546.x 20492295

[23] Liu W, Lu X, He G, Gao X, Xu M, Zhang J, et al Protective roles of Gadd45 and MDM2 in blueberry anthocyanins mediated DNA repair of fragmented and non-fragmented DNA damage in UV-irradiated HepG2 cells. Int J Mol Sci. 2013; 14: 21447–21462. doi: 10.3390/ijms141121447 24177565 10.3390/ijms141121447 PMC3856014

[24] Yi W, Fischer J, Krewer G, Akoh CC. Phenolic compounds from blueberries can inhibit colon cancer cell proliferation and induce apoptosis. J Agric Food Chem. 2005; 53: 7320–7329. doi: 10.1021/jf051333o 16131149 10.1021/jf051333o

[25] Papandreou M, Dimakopoulou A, Linardaki ZI, Cordopatis P, Klimis-Zacas D, Margarity M, et al Effect of polyphenol-rich wild blueberry extract on cognitive performance of mice, brain antioxidant markers and acetylcholinesterase activity. Behav Brain Res. 2009; 198: 352–358. doi: 10.1016/j.bbr.2008.11.013 19056430 10.1016/j.bbr.2008.11.013

[26] Ma C, Dastmalchi K, Flores G, Wu S-B, Pedraza-Peñalosa P, Long C, et al Antioxidant and metabolite profiling of North American and neotropical blueberries using LC-TOF-MS and multivariate analyses. J Agric Food Chem. 2013; 61: 3548–3559. doi: 10.1021/jf400515g 23547798 10.1021/jf400515g

[27] Jensen HD, Krogfelt KA, Cornett C, Hansen SH, Christensen SB. Hydrophilic carboxylic acids and iridoid glycosides in the juice of American and European cranberries (*Vaccinium macrocarpon* and *V*. *oxycoccos*), lingonberries (*V*. *vitis-idaea*), and blueberries (*V*. *myrtillus*). J Agric Food Chem 2002; 50: 6871–6874. 12405790 10.1021/jf0205110

[28] Swiatek L, Komorowski T. The occurance of monotropein and of asperulosdie in some species of the families: Ericaceae, Empetraceae and Rubiaceae. Herba Polonica. 1972; 2: 168–173.

[29] Heffels P, Muller L, Schieber A, Weber F. Profiling of iridoid glycosides in *Vaccinium* species by UHPLC-MS. Food Res Int. 2016; http://dx.doi.org/10.1016/j.foodres.2016.11.018. 10.1016/j.foodres.2016.11.018 28964369

[30] Clark JR, Moore JN ‘Ozarkblue’ southern highbush blueberry. HortScience. 1996; 31: 1043–1045.

[31] Ehlenfeldt MK, Stretch AW. ‘Cara’s Choice’ blueberry. HortScience. 2005; 40: 1556–1557.

