# Supplementary figures and images for: Differential iridoid production as revealed by a diversity panel of 84 cultivated and wild blueberry species
Source: PLoS One. 2017 Jun 13;12(6):e0179417. doi: 10.1371/journal.pone.0179417 (PMC5469490; doi:10.1371/journal.pone.0179417)

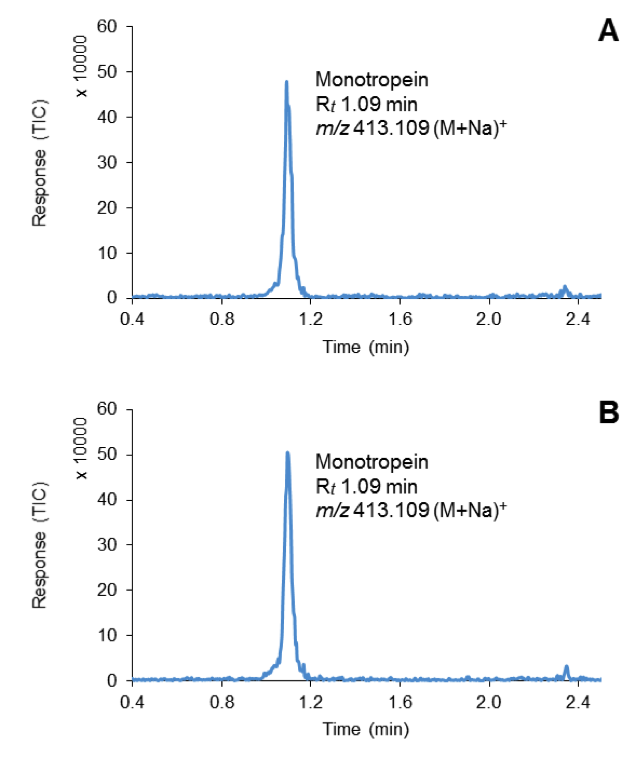

Supplement: S1 Fig — Total ion chromatograms (TICs) of iridoid glycoside, monotropein after one hour incubation in methanol at room temperature (A) and 60°C (B). (TIFF) [file pone.0179417.s001.tiff]
