# Supplementary material for: Differential iridoid production as revealed by a diversity panel of 84 cultivated and wild blueberry species
Source: PLoS One. 2017 Jun 13;12(6):e0179417. doi: 10.1371/journal.pone.0179417 (PMC5469490; doi:10.1371/journal.pone.0179417)
Supplement: S2 Table — (DOCX) [file pone.0179417.s003.docx]

**S2 Table**. The recovery of monotropein standard from a blueberry sample.

| **Sample** | **Extracted sample: Summit** | **Untreated monotropein**  **Standard Added to sample (10 uL of 10 uM to 200 uL)** | **Extracted sample: Summit with monotropein (10 uL of 10 uM) standard added** |
| --- | --- | --- | --- |
| Monotropein peak intensity | 7,676 ± 543 | 14,253 ± 662 | 20,835 ± 577 |
